# Supplementary material for: Expression of Iron Metabolism Proteins in Patients with Chronic Heart Failure
Source: J Clin Med. 2022 Feb 5;11(3):837. doi: 10.3390/jcm11030837 (PMC8837054; doi:10.3390/jcm11030837)
Supplement: Supplementary file 1 [file jcm-11-00837-s001.zip › jcm-1531622-Supplementary.pdf]

Table S1. ELISA kits from ELK Biotechnology Co., Ltd Wuhan, Chine used in the study.

Detected Protein	Catalog Number	Tested Range
divalent metal transporter - (DMT-1)	ELK4762	0.16–10 ng/ml
L-type calcium channel Subunit CACNa1D (L-CH)	ELK3707	0.32–20 ng/ml
transferrin receptor -1(TfR)	ELK2912	0.125–8 ng/ml
transferrin receptor -2 (TfR-2)	ELK2343	0.63–40 ng/ml
ferritin heavy (FT-H),	ELK3136	0.031–2 ng/ml
ferritin—light (FT-L),	ELK3120	0.32–20 ng/ml
ferritin- mitochondrial (FT-MT)	ELK4761	0.32–20 ng/ml
ferroportin (FPN)	ELK3773	0.32–20 ng/ml
hepcidin (Hepc)	ELK5123	0.625–40 ng/ml
aconitase- 1 (ACO-1)	ELK3265	0.63–40 ng/ml
iron response protein-2 (IREB-2)	ELK4764	0.16–10 ng/ml
hypoxia-induced factor (HIF-1)	ELK1603	0.16–10 ng/ml
hemojuvelin (HJV)	ELK5431	0.031–20 ng/ml
4-hydroxynonenal (4-HNE)	ELK8372	0.039–2.5 ng/ml

Table S2. A-B. Comprehensive clinical characteristics of the study group. A—demographic information, laboratory tests, echocardiography parameters, hemodynamic parameters, levels of iron-metabolism markers. B– concomitant treatment, electrotherapy, other data. Me—median. Q—quartile; N—number of patients, p—level of significance; **bold**—significant differences.

A

	**unit**	**Heart Failure**		**Women**		**Men**		***p*-Value**
		**Me**	**Q**	**Me**	**Q**	**Me**	**Q**	
**Demographic information**								
Age	Yrs	53.5	10.5	58.0	10.0	53.0	10.5	0.4728
Height	Cm	175.5	6.75	164	3.5	178	5	**0.0001**
Body weight	Kg	80.0	10.5	61	11.5	82.5	9.5	**0.0005**
BSA- Body Surface Area	m2	2.0	0.2	1.7	0.2	2.0	0.1	**0.0001**
BMI- Body-mass index	kg/m2	25.6	3.3	22.8	4.1	25.9	2.6	0.1453
**Laboratory tests**								
NTproBNP	pg/ml	3955.5	2250.5	4436	1291.5	3833	2665	0.4014
CRP	mg/dl	0.4	0.3	0.3	0.2	0.5	0.3	0.5100
Na	mmol/l	138.0	3.0	139.0	3.0	138.0	3.0	0.1357
K	mmol/l	4.3	0.3	4.3	0.3	4.3	0.3	0.5200
Creatinine	mg/dl	1.2	0.2	1.0	0.1	1.2	0.1	**0.0376**
Glomerular filtration rate	ml/min/1.73m2	60.0	4.5	60.0	5.0	60.0	4.3	0.5569
Urea	mg/dl	47.0	9.9	45.6	9.6	47.0	10.0	0.8640
AST	U/I	26.5	7.5	25.0	8.5	27	6.0	0.2184
ALT	U/I	25.0	11.5	19.0	3.0	27.0	10.0	**0.0218**
Bilirubin	mg/dl	0.7	0.4	1.0	0.2	0.6	0.3	0.3184
Protein	g/dl	7.1	0.48	7.24	0.7	7.1	0.4	0.7231
Glucose	mg/dl	93.78	9.9	98	11.93	91.9	8.82	0.2128
Uric acid	mg/dl	7.3	1.269155	7.7	1.52162	7.3	1.235535	0.5237
Total cholesterol	mg/dl	145.8	26.5	148.8	10.5	144.6	33	0.5918
LDL	mg/dl	94	22.5	105.4	25	89.1	23.7	0.2385
HDL	mg/dl	34.61	8.62	34.23	7	35	8.62	0.9034
TG	mg/dl	107	23.25	103	15	108	31.75	0.5802

	unit	Heart Failure		Women		Men		*p*-Value
		**Me**	**Q**	**Me**	**Q**	**Me**	**Q**	
				**Echocardiography parameters**				
LVED	mm	67	8	58.5	10.5	70	8.5	**0.0069**
LVSD	mm	59	13	43	10.5	65	12	**0.0033**
LVEF	%	20	7.5	20	10	20	5.5	0.1811
PW	mm	10	1.5	7.5	1.75	10	1.5	**0.0099**
IVS	mm	9.15	2	9	1.9	9.3	2	0.2365
PA	mm	24	3.5	22.5	1.875	27	3	**0.0310**
RV	mm	42	6.5	42	11	42	5.5	0.8857
IM	+/++++	1.5	0.5	1.5	0.5	1.5	0.5	0.8520
IT	+/++++	1.75	0.75	2.5	0.5	1.5	0.5	**0.0184**
PASP	mmHg	60	10	68.5	19	57.5	10	0.3490
RVSP	mmHg	47	12.5	45	12.5	47	11	0.6869
AccT	ms	87.5	17	87	21.5	87.5	17	1
VTI LVOT	cm	10.4	4.1	12	0	9.7	5.8	1
TAPSE	mm	15	2	13	2	16	2	**0.0133**
				**Hemodynamic parameters**				
RAP	mmHg	12	4.5	11.5	7.25	12	4.5	0.9026
PAPs	mmHg	42	12.5	36	8.5	44	12	0.1052
PAPd	mmHg	21	5	22.5	6.5	21	5	0.8147
PAPm	mmHg	29	7.5	26.5	5.5	29	7	0.2711
PWPm	mmHg	21	5.5	19.	19.5	21	7.5	0.2624
TCG	mmHg	8.5	3	9	1.5	8	3	0.4742
CO	l/min	3.45	0.775	2.56	0.3675	3.7	0.675	0.0058
PVR	Wood Units	2.21	0.93	2.73	1.3575	2.21	0.73	0.2157
SVR	Wood Units	13.3	2.795	22.38	10.85	12.6	2.2475	0.0969
sBP	mmHg	104.5	13	111	14	104	13	1
dBP	mmHg	65.5	5.5	65.5	7.5	65.5	5.5	0.9806
mBP	mmHg	78	7.5	82	8	76	7	0.6020
				**Levels of Iron-Metabolism Markers**				
RBC	10^6/ul	4.54	0.51	4.77	0.51	4.54	0.475	0.1950

	unit	Heart Failure		Women		Men		*p*-Value
		Me	Q	Me	Q	Me	Q	
HT	%	42	4.3	43.4	5.3	42	4.5	0.2964
Hb	g/dl	14.05	1.4	14.3	1.5	14	1.2	0.5261
MCV	fl	91	3.55	90	4.8	91	3.5	0.7371
MCH	pg	30.6	1.5	30.9	1.3	30.3	1.5	0.8012
MCHC	pg	32.9	1	32.65	0.67	33.5	0.95	0.1193
RDW-SD	fl	47.8	3.95	48.1	4.9	47.6	3.75	0.6845
RDW-CV	%	14.4	1.4	14.35	1.22	14.5	1.4	0.3431
WBC	10^3/ul	7.6	1.25	6.6	1.05	7.7	1.2	0.0676
PLT	10^3/ul	202	44	200	39.5	204	44	0.9925
MPV	fl	11	0.7	10.2	0.57	11.2	0.75	0.0709
RET %	%	1.635	0.495	1.69	0.235	1.58	0.53	0.7642
RET	10^6/ul	0.067	0.0185	0.076	0.007	0.0655	0.019825	0.4549
IRF	%	15.45	5.65	16.9	7.9	14.7	5.55	0.2399
LRF	%	85.3	4.6	83.1	7.9	85.6	5.05	0.2088
MFR	%	11.4	2.4	14.4	1.55	11.3	3.25	0.2088
HFR	%	2.95	2.6	3	5.65	2.9	2.15	0.3937
RET-Hb	pg	32	2	26	3.1	32	1.575	0.0960
Iron	ug/dl	77.55	27.15	79.1	25	76	29.7	0.5820
Ferritin	ng/ml	212.1	89.1	230	122.5	202.55	82.5	0.8663
Transferrin	mg/dl	242	51.5	70.785	130.675	246	43.5	0.0645
TSAT	%	21	8.55	18	7.1	24.2	7.5	0.1138
sTFR	mg/l	3.185	1.14	3.26	1.565	3.185	1.21	0.4380
sTfR1/logFR ferritin	-	0.61	0.27	0.63	0.36	0.61	0.21	0.2327
Erythropoietin	mIU/ml	18.3	11.35	28.55	16.125	16.1	10.11	**0.0386**
Folic acid	ng/ml	7.45	2.0475	8.4	1.895	7.34	2.005	0.4040
Vitamin B12	pg/ml	395.3	137.15	386	80.1	396.5	148.05	0.61343

Aberrations: NTproBNP (N-terminal pro-brain natriuretic peptide), CRP (C-reactive protein), AST (aspartate aminotransferase), ALT (alanine aminotransferase), LDL (low-density lipoprotein), HDL (high-density lipoprotein), TG (triglycerides), LVED (left ventricle diameters diastolic), LVSD (left ventricle diameters systolic), LVEF(left ventricle ejection fraction), PW (posterior wall), IVS (intraventricular septal width), PA (pulmonary artery), RV (right ventricle diastolic size), IM (mitral insufficiency), IT(tricuspid insufficiency), PASP (pulmonary artery pressure systolic),), RVSP (right ventricular systolic pressure), AccT (acceleration time), VTI LVOT (Left ventricular outflow tract velocity time integral), TAPSE (tricuspid annular plane systolic excursion), RAP (right atrium pressure), PAPs (pulmonary artery pressure systolic), PAPm (pulmonary artery pressure mean), PWPm (pulmonary capillary wedge pressure mean), TCG (transpulmonary

pressure gradient), CO(cardiac output), PVR (pulmonary vascular resistance), SVR (systemic vascular resistance), sBP (systolic blood pressure), dBP (diastolic blood pressure), mBP (mean blood pressure), RBC (red blood cells), HT (hematocrit), Hb (hemoglobin), MCV (mean corpuscular volume), MCH (mean corpuscular hemoglobin), MCHC (mean corpuscular hemoglobin concentration), RDW-SD (red blood cell distribution width-standard deviation), RDW-CV (red blood cell distribution width- coefficient of variation), WBC (white blood cells), PLT (platetes), MPV (mean platelet volume), RET % (reticulocyte %),RET (reticulocute), IRF (immature reticulocyte fraction), LRF (low-fluorescence reticulocytes.), MFR(middle-fluorescence reticulocytes....), HFR (high-fluorescence reticulocytes), RET-Hb (reticulocyte hemoglobin), TSAT (transferrin saturation), sTfR (soluble transferrin receptor) sTfR1/logFR (soluble transferrin receptor/logarithm of ferritin).

B

	Heart Failure		Women		Men		P-Value
	N	%	N	%	N	%	
Concomitant treatment							
ACE inhibitor or ARB	49	84.5	11	84.6	38	84.44	
ARNI	2	3.4	0	0	2	15.38	
MRI	51	87.9	11	84.6	40	88.89	
Beta-blocker	55	94.8	13	100	42	93.33	
Digitalis glycoside	12	20.7	3	23.08	9	20.00	
Ivabradine	8	1.4	2	15.38	6	13.33	
Amiodarone	11	19	1	7.69	10	22.22	
Diuretic	53	91.4	11	84.6	42	93.33	
Lipid-lowering therapy	30	51.7	7	18.57	23	51.11	
Antiplatelet therapy	20	34.5	3	23.07	17	37.78	
Vitamin k antagonist/ novel oral anticoagulants	37	63.8	10	76.92	27	60.00	
Allopurinol	22	37.9	4	30.76	18	40.00	
Dopamine	8	13.8	4	30.76	4	8.89	
Dobutamine	19	32.7	4	30.76	15	33.33	
Norepinephrine	3	5.2	0	0	3	6.67	
Supplementation of thyroid hormones	11	19	5	38.46	6	13.33	
Hyperthyroidism drugs	1	1.6	1	7.69	0	0	
Antidiabetic drugs	6	10.3	2	15.38	4	8.89	
Sildenafil	6	10.3	1	7.69	5	11.1	
Electrotherapy							

	Heart Failure		Women		Men		P-Value
	**N**	**%**	**N**	**%**	**N**	**%**	
Implantable cardioverter-defibrillator- ICD	10	17.2	4	30.76	6	13.33	
Cardiac resynchronization therapy- CRT-D	35	60.4	8	61.54	27	60	
			Other data				
Ischemic	18.0	31.0	2.0	15.4	16.0	35.6	
Cardiomyopathy	36.0	62.1	9.0	69.2	27.0	60	0.2008
Other	4.0	6.9	2	15.4	2	44.4	
NYHA: 3	31	53.4	6	46.2	25	55.5	0.5494
NYHA: 4	27	46.6	7	53.84	20	44.4	
Sinus rhythm	40	69	7	53.84	33	73.3	0.1810
No-Sinus rhythm	18	31	6	46.2	12	26.7	

Table S3. Characteristics of the control group. Me—median. Q—quartile.

**Demographic Information**	**Control group**	**n = 31**
	**Me**	**Q**
Age	36.0	10.0
Women	n = 11	35.5%
Men	n = 20	64.5%
Height	175.0	7.0
Body weight	77.0	11.5
BSA- Body Surface Area	1.9	0.2
BMI- Body-mass index	24.8	2.6

Table S4. Expression of iron handling proteins, iron regulatory factors, and an oxidative stress marker in the non-failing compared to the failing human myocardium. P—level of significance; **bold**—significant differences.

Parameter	Units	Non- Failing heart	Failing Heart	*p*-value	Failing Heart		*p*-value
					Women	Men	
DMT-1	ng/ml	**5.8 +/- 1.1**	**4.3 +/- 0.6**	**0.0001**	4.3 +/- 0.7	4.3 +/- 0.6	0.067654
L-CH	ng/ml	**67.3 +/- 23.2**	**47.3 +/- 8.5**	**0.0019**	42.8 +/- 7.3	48.4 +/- 8.1	0.709208
TfR-1	ng/ml	**173.0 +/- 36.5**	**152.0 +/- 30.8**	**0.0038**	155.3 +/- 24.8	151.6 +/- 33.4	0.635486
TfR-2	ng/ml	11.9 +/- 1.2	11.3 +/- 2.1	0.1541	12.4 +/- 1.5	11.1 +/- 2.0	0.68165
FT-H	ng/ml	**28.1 +/- 5.0**	**23.5 +/- 3.8**	**0.0189**	24.3 +/- 3.2	23.4 +/- 3.6	0.411974
FT-L	ng/ml	**22.1 +/- 7.4**	**15.0 +/- 5.7**	**0.0001**	17.1 +/- 4.6	14.3 +/- 5.3	0.723133
FT-MT	ng/ml	**55.9 +/- 12.4**	**39.1 +/- 6.1**	**0.0001**	37.7 +/- 5.2	40.1 +/- 6.1	0.17943
FPN	ng/ml	75.4 +/- 14.6	80.5 +/- 28.0	0.3199	89.9 +/- 37.0	76.4 +/- 26.1	0.24766
Hepc	ng/ml	**23.8 +/- 7.2**	**18.7 +/- 6.0**	**0.0812**	24.9 +/- 9.1	17.9 +/- 4.6	0.082904
ACO-1	ng/ml	**58.1 +/- 8.6**	**50.0 +/- 5.4**	**0.0045**	54.7 +/- 3.9	49.7 +/- 6.0	0.444577
IREB-2	ng/ml	**1.0 +/- 0.3**	**0.7 +/- 0.1**	**0.0104**	0.7 +/- 0.1	0.7 +/- 0.1	0.542224
HIF- 1	ng/ml	**11.8 +/- 4.2**	**7.9 +/- 2.2**	**0.0007**	10.0 +/- 2.4	7.3 +/- 1.9	0.925721
HJV	ng/ml	47.8 +/- 17.4	48.7 +/- 9.6	0.8262	46.5 +/- 9.5	49.2 +/- 9.6	0.68165
4-HNE	pg/ml	**10.6 +/- 8.4**	**55.7 +/- 33.7**	**0.0001**	54.8 +/- 20.6	57.1 +/- 36.2	0.156455

Aberrations: DMT-1 (divalent metal transporter -1), L-CH (L-type calcium channel Subunit CACNa1D), TfR-1 or -2 (transferrin receptor -1 or -2), FT-H (ferritin heavy chain), FT-L (ferritin light chain), FT-MT (ferritin- mitochondrial), FPN (ferroportin), Hepc (hepcidin), ACO-1 (aconitase- 1), IREB-2 (iron response protein-2), HIF-1 (hypoxia-induced factor), HJV (hemojuvelin), 4-HNE (4-hydroxynonenal).

Table S5. Expression of iron handling proteins, iron regulatory factors, and an oxidative stress marker in the non-failing compared to the failing human myocardium with or without iron deficiency. [a ; b]—levels of significance.

Parameter	Units	Non- Failing Heart n = 31	Failing Heart Non-Iron Deficient n = 26	Failing Heart Iron Deficient n = 32
DMT-1	ng/ml	5.84 ± 1.07 a	4.18 ± 0.47 b	4.36 ± 0.56 b
L-CH	ng/ml	67.33 ± 23.19 a	43.08 ± 7.43 b	54.03 ± 7.57 ab
TfR-1	ng/ml	173.02 ± 36.54 a	142.72 ± 32.17 b	153.51 ± 31.22 ab
TfR-2	ng/ml	11.88 ± 1.24	11.14 ± 1.21	11.28 ± 2.25
FT-H	ng/ml	28.07 ± 4.98	24.03 ± 3.26	22.44 ± 5.47
FT-L	ng/ml	22.08 ± 7.41 a	17.03 ± 6.53 b	12.24 ± 5.49 b
FT-MT	ng/ml	55.94 ± 12.43 a	34.77 ± 6.04 b	44.05 ± 4.36 b
FPN	ng/ml	75.40 ± 14.58	69.80 ± 24.95	82.99 ± 27.27

Aberrations: DMT-1 (divalent metal transporter -1), L-CH (L-type calcium channel Subunit CACNa1D), TfR-1 or -2 (transferrin receptor -1 or -2), FT-H (ferritin heavy chain), FT-L (ferritin light chain), FT-MT (ferritin- mitochondrial), FPN (ferroportin).

Table S6. Correlations between iron handling proteins, iron regulatory factors, and an oxidative stress marker in the non-failing human myocardium. X—not analyzed; * - n = 31; ° - n = 26; p—level of significance; **bold**—significant differences.

	**DMT-1***	**L-CH°**	**TfR-1***	**TfR-2***	**FT-H***	**FT-L***	**FT-MT***	**FPN***	**Hepc***	**ACO-1***	**IREB-2***	**HIF- 1***	**HJV***	**4-HNE°**
**DMT-1***	X	0.3451<br>*p* = 0.084	0.1619<br>*p* = 0.384	0.0335<br>*p* = 0.858	0.1290<br>*p* = 0.489	0.1435<br>*p* = 0.441	**0.3936**<br>***p* = 0.028**	0.1820<br>*p* = 0.327	0.1202<br>*p* = 0.519	0.0855<br>*p* = 0.647	0.2264<br>*p* = 0.221	-0.0680<br>*p* = 0.716	-0.1741<br>*p* = 0.349	-0.1057<br>*p* = 0.607
**L-CH**	0.3451<br>*p* = 0.084	X	**0.5289**<br>***p* = 0.005**	0.3672<br>*p* = 0.065	0.3441<br>*p* = 0.085	**0.7371**<br>***p* = 0.000**	**0.6245**<br>***p* = 0.001**	**0.5302**<br>***p* = 0.005**	0.0442<br>*p* = 0.830	0.3753<br>*p* = 0.059	**0.5198**<br>***p* = 0.007**	0.2674<br>*p* = 0.187	**0.4088**<br>***p* = 0.038**	-0.2757<br>*p* = 0.173
**TfR-1***	0.1619<br>*p* = 0.384	**0.5289**<br>***p* = 0.005**	X	-0.0163<br>*p* = 0.931	**0.4022**<br>***p* = 0.025**	-0.1671<br>*p* = 0.369	0.1129<br>*p* = 0.546	**0.4634**<br>***p* = 0.009**	-0.1610<br>*p* = 0.387	**0.3809**<br>***p* = 0.034**	**0.6008**<br>***p* = 0.000**	-0.3222<br>*p* = 0.077	0.2939<br>*p* = 0.109	**-0.4596**<br>***p* = 0.018**
**TfR-2***	0.0335<br>*p* = 0.858	0.3672<br>*p* = 0.065	-0.0163<br>*p* = 0.320	X	0.1431<br>*p* = 0.442	0.0026<br>*p* = 0.989	-0.0645<br>*p* = 0.730	0.0761<br>*p* = 0.684	0.0163<br>*p* = 0.931	0.1845<br>*p* = 0.320	-0.2211<br>*p* = 0.320	0.0727<br>*p* = 0.698	0.3406<br>*p* = 0.061	0.2743<br>*p* = 0.175
**FT-H***	0.1290<br>*p* = 0.489	0.3441<br>*p* = 0.085	**0.4022**<br>***p* = 0.025**	0.1431<br>*p* = 0.442	X	0.1111<br>*p* = 0.552	0.2337<br>*p* = 0.206	**0.6595**<br>***p* = 0.000**	**0.4007**<br>***p* = 0.025**	**0.4951**<br>***p* = 0.005**	**0.4660**<br>***p* = 0.008**	-0.0774<br>*p* = 0.679	0.0802<br>*p* = 0.668	-0.3205<br>*p* = 0.110
**FT-L***	0.1435<br>*p* = 0.441	**0.7371**<br>***p* = 0.000**	-0.1671<br>*p* = 0.369	0.0026<br>*p* = 0.989	0.1111<br>*p* = 0.552	X	**0.6626**<br>***p* = 0.000**	0.0283<br>*p* = 0.880	0.3427<br>*p* = 0.059	0.2516<br>*p* = 0.172	0.1147<br>*p* = 0.539	0.3326<br>*p* = 0.067	0.2321<br>*p* = 0.209	-0.2119<br>*p* = 0.299
**FT-MT***	**0.3936**<br>***p* = 0.028**	**0.6245**<br>***p* = 0.001**	0.1129<br>*p* = 0.546	-0.0645<br>*p* = 0.730	0.2337<br>*p* = 0.206	**0.6626**<br>***p* = 0.000**	X	0.2352<br>*p* = 0.203	0.3535<br>*p* = 0.051	0.1324<br>*p* = 0.478	**0.4145**<br>***p* = 0.020**	**0.3937**<br>***p* = 0.028**	0.0366<br>*p* = 0.845	**-0.4148**<br>***p* = 0.035**
**FPN**	0.1820<br>*p* = 0.327	**0.5302**<br>***p* = 0.005**	0.4634<br>*p* = 0.009	0.0761<br>*p* = 0.684	**0.6595**<br>***p* = 0.000**	0.0283<br>*p* = 0.880	0.2352<br>*p* = 0.203	X	0.1572<br>*p* = 0.398	0.2684<br>*p* = 0.144	**0.6483**<br>***p* = 0.000**	0.0578<br>*p* = 0.758	-0.1470<br>*p* = 0.430	**-0.5976**<br>***p* = 0.001**
**Hepc***	0.1202<br>*p* = 0.519	0.0442<br>*p* = 0.830	-0.1610<br>*p* = 0.387	0.0163<br>*p* = 0.931	**0.4007**<br>***p* = 0.025**	0.3427<br>*p* = 0.059	0.3535<br>*p* = 0.051	0.1572<br>*p* = 0.398	X	0.2607<br>*p* = 0.157	0.2012<br>*p* = 0.278	**0.4282**<br>***p* = 0.016**	0.0112<br>*p* = 0.952	-0.1459<br>*p* = 0.477
**ACO-1***	0.0855<br>*p* = 0.647	0.3753<br>*p* = 0.059	**0.3809**<br>***p* = 0.034**	0.1845<br>*p* = 0.320	**0.4951**<br>***p* = 0.005**	0.2516<br>*p* = 0.172	0.1324<br>*p* = 0.478	0.2684<br>*p* = 0.144	0.2607<br>*p* = 0.157	X	0.3242<br>*p* = 0.075	0.0060<br>*p* = 0.974	**0.4390**<br>***p* = 0.013**	-0.1669<br>*p* = 0.415
**IREB-2***	0.2264	**0.5198**	**0.6008**	-0.2211	**0.4660**	0.1147	**0.4145**	**0.6483**	0.2012	0.3242	X	0.0170	-0.1992	**-0.5620**

	DMT-1*	L-CH°	TfR-1*	TfR-2*	FT-H*	FT-L*	FT-MT*	FPN*	Hepc*	ACO-1*	IREB-2*	HIF- 1*	HJV*	4-HNE°
	p = 0.221	***p* = 0.007**	***p* = 0.000**	*p* = 0.232	***p* = 0.008**	*p* = 0.539	***p* = 0.020**	***p* = 0.000**	*p* = 0.278	*p* = 0.075		*p* = 0.928	*p* = 0.283	***p* = 0.003**
**HIF- 1***	-0.0680	0.2674	-0.3222	0.0727	-0.0774	0.3326	**0.3937**	0.0578	**0.4282**	0.0060	0.0170	X	-0.0397	-0.1829
	p = 0.716	*p* = 0.187	*p* = 0.077	*p* = 0.698	*p* = 0.679	*p* = 0.067	***p* = 0.028**	*p* = 0.758	***p* = 0.016**	*p* = 0.974	*p* = 0.928		*p* = 0.832	*p* = 0.371
**HJV***	-0.1741	**0.4088**	0.2939	0.3406	0.0802	0.2321	0.0366	-0.1470	0.0112	**0.4390**	-0.1992	-0.0397	X	0.2536
	p = 0.349	***p* = 0.038**	*p* = 0.109	*p* = 0.061	*p* = 0.668	*p* = 0.209	*p* = 0.845	*p* = 0.430	*p* = 0.952	***p* = 0.013**	*p* = 0.283	*p* = 0.832		*p* = 0.211
**4-HNE°**	-0.1057	-0.2757	**-0.4596**	0.2743	-0.3205	-0.2119	**-0.4148**	**-0.5976**	-0.1459	-0.1669	**-0.5620**	-0.1829	0.2536	X
	p = 0.607	*p* = 0.173	***p* = 0.018**	*p* = 0.175	*p* = 0.110	*p* = 0.299	***p* = 0.035**	***p* = 0.001**	*p* = 0.477	*p* = 0.415	***p* = 0.003**	*p* = 0.371	*p* = 0.211	

Aberrations: DMT-1 (divalent metal transporter -1), L-CH (L-type calcium channel Subunit CACNa1D), TfR-1 or -2 (transferrin receptor -1 or -2), FT-H (ferritin heavy chain), FT-L (ferritin light chain), FT-MT (ferritin- mitochondrial), FPN (ferroportin), Hepc (hepcidin), ACO-1 (aconitase- 1), IREB-2 (iron response protein-2), HIF-1 (hypoxia-induced factor), HJV (hemojuvelin), 4-HNE (4-hydroxynonenal).

Table S7. Correlations between iron handling proteins, iron regulatory factors, and an oxidative stress marker in the failing human myocardium. * - n = 31; [#] - n = 58; p—level of significance; **bold**—significant differences.

	DMT-1	L-CH*	TfR-1 [#]	TfR-2 [#]	FT-H [#]	FT-L [#]	FT-MT [#]	FPN [#]	Hepc [#]	ACO-1 [#]	IREB-2 [#]	HIF- 1 [#]	HJV [#]	4-HNE*
**DMT-1 [#]**	X	**0.6545**<br>**p = 0.000**	**0.4456**<br>**p = 0.000**	0.2167<br>*p* = 0.102	**0.2854**<br>**p = 0.030**	**0.2842**<br>**p = 0.031**	**0.3407**<br>**p = 0.009**	**0.3426**<br>**p = 0.008**	0.0061<br>*p* = 0.964	**0.3624**<br>**p = 0.005**	**0.4261**<br>**p = 0.001**	**0.5123**<br>**p = 0.000**	**0.3104**<br>**p = 0.018**	**-0.3773**<br>**p = 0.036**
**L-CH***	**0.6545**<br>**p = 0.000**	X	**0.3954**<br>**p = 0.028**	0.1794<br>*p* = 0.334	**0.4309**<br>**p = 0.016**	**0.4909**<br>**p = 0.005**	-0.0017<br>*p* = 0.993	**0.5214**<br>**p = 0.003**	**-0.4626**<br>**p = 0.009**	**0.3894**<br>**p = 0.030**	0.2949<br>*p* = 0.107	**0.5682**<br>**p = 0.001**	**0.4971**<br>**p = 0.004**	**-0.5707**<br>**p = 0.001**
**TfR-1 [#]**	**0.4456**<br>**p = 0.000**	**0.3954**<br>**p = 0.028**	X	-0.007<br>*p* = 0.958	**0.3627**<br>**p = 0.005**	0.1107<br>*p* = 0.408	0.1986<br>*p* = 0.135	**0.3804**<br>**p = 0.003**	-0.1849<br>*p* = 0.165	0.2545<br>*p* = 0.054	**0.2644**<br>**p = 0.045**	0.13<br>*p* = 0.331	**0.5472**<br>**p = 0.000**	-0.3112<br>*p* = 0.088
**TfR-2 [#]**	0.2167<br>*p* = 0.102	0.1794<br>*p* = 0.334	-0.007<br>*p* = 0.958	X	0.0274<br>*p* = 0.838	**0.4116**<br>**p = 0.001**	**0.2916**<br>**p = 0.026**	0.1147<br>*p* = 0.391	0.0181<br>*p* = 0.893	0.1673<br>*p* = 0.209	0.1882<br>*p* = 0.157	0.1199<br>*p* = 0.370	-0.1709<br>*p* = 0.200	0.0532<br>*p* = 0.776
**FT-H [#]**	**0.2854**<br>**p = 0.030**	**0.4309**<br>**p = 0.016**	**0.3627**<br>**p = 0.005**	0.0274<br>*p* = 0.838	X	**0.5119**<br>**p = 0.000**	0.2335<br>*p* = 0.078	0.0847<br>*p* = 0.527	-0.1326<br>*p* = 0.321	0.2247<br>*p* = 0.090	**0.2815**<br>**p = 0.032**	0.2337<br>*p* = 0.077	0.0633<br>*p* = 0.637	**-0.379**<br>**p = 0.036**
**FT-L [#]**	**0.2842**<br>**p = 0.031**	**0.4909**<br>**p = 0.005**	0.1107<br>*p* = 0.408	**0.4116**<br>**p = 0.001**	**0.5119**<br>**p = 0.000**	X	0.001<br>*p* = 0.994	0.0442<br>*p* = 0.742	0.0397<br>*p* = 0.767	-0.0219<br>*p* = 0.871	-0.145<br>*p* = 0.278	**0.2597**<br>**p = 0.049**	0.1399<br>*p* = 0.295	-0.2013<br>*p* = 0.278
**FT-MT [#]**	**0.3407**<br>**p = 0.009**	-0.0017<br>*p* = 0.993	0.1986<br>*p* = 0.135	**0.2916**<br>**p = 0.026**	0.2335<br>*p* = 0.078	0.001<br>*p* = 0.994	X	0.005<br>*p* = 0.970	-0.1229<br>*p* = 0.358	**0.3277**<br>**p = 0.012**	**0.5455**<br>**p = 0.000**	**0.3648**<br>**p = 0.005**	-0.1648<br>*p* = 0.216	0.2483<br>*p* = 0.178
**FPN [#]**	**0.3426**<br>**p = 0.008**	**0.5214**<br>**p = 0.003**	**0.3804**<br>**p = 0.003**	0.1147<br>*p* = 0.391	0.0847<br>*p* = 0.527	0.0442<br>*p* = 0.742	0.005<br>*p* = 0.970	X	**-0.2833**<br>**p = 0.031**	0.247<br>*p* = 0.062	0.2062<br>*p* = 0.120	0.2231<br>*p* = 0.092	0.2034<br>*p* = 0.126	**-0.4219**<br>**p = 0.018**
**Hepc [#]**	0.0061<br>*p* = 0.964	**-0.4626**<br>**p = 0.009**	-0.1849<br>*p* = 0.165	0.0181<br>*p* = 0.893	-0.1326<br>*p* = 0.321	0.0397<br>*p* = 0.767	-0.1229<br>*p* = 0.358	**-0.2833**<br>**p = 0.031**	X	-0.2314<br>*p* = 0.081	-0.165<br>*p* = 0.216	0.1756<br>*p* = 0.187	-0.0367<br>*p* = 0.785	-0.0907<br>*p* = 0.628
**ACO-1 [#]**	**0.3624**<br>**p = 0.005**	**0.3894**<br>**p = 0.030**	0.2545<br>*p* = 0.054	0.1673<br>*p* = 0.209	0.2247<br>*p* = 0.090	-0.0219<br>*p* = 0.871	**0.3277**<br>**p = 0.012**	0.247<br>*p* = 0.062	-0.2314<br>*p* = 0.081	X	**0.5804**<br>**p = 0.000**	**0.2639**<br>**p = 0.045**	0.0837<br>*p* = 0.532	**-0.4478**<br>**p = 0.012**
**IREB-2 [#]**	**0.4261**<br>**p = 0.001**	0.2949<br>*p* = 0.107	**0.2644**<br>**p = 0.045**	0.1882<br>*p* = 0.157	**0.2815**<br>**p = 0.032**	-0.145<br>*p* = 0.278	**0.5455**<br>**p = 0.000**	0.2062<br>*p* = 0.120	-0.165<br>*p* = 0.216	**0.5804**<br>**p = 0.000**	X	0.2266<br>*p* = 0.087	-0.1181<br>*p* = 0.377	-0.3355<br>*p* = 0.065
**HIF- 1**	**0.5123**<br>**p = 0.000**	**0.5682**<br>**p = 0.001**	0.13<br>*p* = 0.331	0.1199<br>*p* = 0.370	0.2337<br>*p* = 0.077	**0.2597**<br>**p = 0.049**	**0.3648**<br>**p = 0.005**	0.2231<br>*p* = 0.092	0.1756<br>*p* = 0.187	**0.2639**<br>**p = 0.045**	0.2266<br>*p* = 0.087	X	0.1402<br>*p* = 0.294	-0.3403<br>*p* = 0.061
**HJV [#]**	**0.3104**<br>**p = 0.018**	**0.4971**<br>**p = 0.004**	**0.5472**<br>**p = 0.000**	-0.1709<br>*p* = 0.200	0.0633<br>*p* = 0.637	0.1399<br>*p* = 0.295	-0.1648<br>*p* = 0.216	0.2034<br>*p* = 0.126	-0.0367<br>*p* = 0.785	0.0837<br>*p* = 0.532	-0.1181<br>*p* = 0.377	0.1402<br>*p* = 0.294	X	-0.2766<br>*p* = 0.132
**4-HNE***	**-0.3773**<br>**p = 0.036**	**-0.5707**<br>**p = 0.001**	-0.3112<br>*p* = 0.088	0.0532<br>*p* = 0.776	**-0.379**<br>**p = 0.036**	-0.2013<br>*p* = 0.278	0.2483<br>*p* = 0.178	**-0.4219**<br>**p = 0.018**	-0.0907<br>*p* = 0.628	**-0.4478**<br>**p = 0.012**	-0.3355<br>*p* = 0.065	-0.3403<br>*p* = 0.061	-0.2766<br>*p* = 0.132	X

Aberrations: DMT-1 (divalent metal transporter -1), L-CH (L-type calcium channel Subunit CACNa1D), TfR-1 or -2 (transferrin receptor -1 or -2), FT-H (ferritin heavy chain), FT-L (ferritin light chain), FT-MT (ferritin- mitochondrial), FPN (ferroportin), Hepc (hepcidin), ACO-1 (aconitase- 1), IREB-2 (iron response protein-2), HIF-1 (hypoxia-induced factor), HJV (hemojuvelin), 4-HNE (4-hydroxynonenal).

Table S8. Correlations between iron-handling proteins in the failing human myocardium, iron serum markers and heart failure parameters.* -

n = 31; - n = 58; p—level of significance; **bold**—significant differences.

	Hb	Ferritin	sTfR	TSAT	CRP	LVEF	TCG	CO	NT-proBNP
**DMT-1***	0.1043	0.1269	0.2693	0.0620	0.1027	0.0440	,21130.	0.0392	-0.1896
	p = 0.342	*p* = 0.338	*p* = 0.059	*p* = 0.659	*p* = 0.551	*p* = 0.741	*p* =0.129	*p* = 0.774	*p* = 0.140
**L-CH**	-0.0788	0.1675	0.0708	0.2131	0.1324	0.0620	0.1318	0.2545	0.0538
	p = 0.0556	*p* = 0.336	*p* = 0.705	*p* = 0.226	*p* = 0.463	*p* = 0.736	*p* = 0.488	*p* = 0.167	*p* = 0.759
**TfR-1***	0.0843	0.1670	0.2690	-0.0217	0.1645	0.1216	0.2299	**0.2646**	-0.0115
	p = 0.443	*p* = 0.206	*p* = 0.059	*p* = 0.878	*p* = 0.338	*p* = 0.359	*p* = 0.222	**p = 0.049**	*p* = 0.929
**TfR-2***	0.1533	0.1443	0.2067	-0.0563	0.1256	0.0016	0.3259	0.0407	0.1171
	p = 0.161	*p* = 0.276	*p* = 0.150	*p* = 0.689	*p* = 0.465	*p* = 0.990	*p* = 0.079	*p* = 0.766	*p* = 0.365
**FT-H***	-0.0256	**0.3725**	-0.0382	0.0714	0.0415	0.0039	0.2732	0.0700	0.0573
	p = 0.816	**p = 0.004**	*p* = 0.792	*p* = 0.612	*p* = 0.810	*p* = 0.977	*p* = 0.144	*p* = 0.608	*p* = 0.658
**FT-L***	-0.0836	**0.4329**	-0.0519	0.0047	0.0837	-0.1396	0.3101	-0.1657	0.1382
	p = 0.447	**p = 0.001**	*p* = 0.720	*p* = 0.973	*p* = 0.628	*p* = 0.292	*p* = 0.095	*p* = 0.222	*p* = 0.284
**FT-MT***	-0.1012	-0.0974	**0.3424**	-0.0015	0.2698	0.1714	0.0382	**0.2743**	-0.0024
	p = 0.357	*p* = 0.463	**p = 0.015**	*p* = 0.992	*p* = 0.111	*p* = 0.194	*p* = 0.841	**p = 0.041**	*p* = 0.985
**FPN**	0.0598	0.2274	0.1950	-0.0423	0.1323	0.0662	0.1130	0.1815	0.0673
	p = 0.587	*p* = 0.083	*p* = 0.175	*p* = 0.764	*p* = 0.442	*p* = 0.618	*p* = 0.552	*p* = 0.181	*p* = 0.603

Aberrations: DMT-1 (divalent metal transporter -1), L-CH (L-type calcium channel Subunit CACNa1D), TfR-1 or -2 (transferrin receptor -1 or -2), FT-H (ferritin heavy chain), FT-L (ferritin light chain), FT-MT (ferritin- mitochondrial), FPN (ferroportin), Hepc (hepcidin), Hb (hemoglobin), sTfR (soluble transferrin receptor), TSAT (transferrin saturation), CRP (C-reactive protein), LVEF(left ventricle ejection fraction), TCG (transpulmonary pressure gradient), CO (cardiac output), NT-proBNP (N-terminal pro-brain natriuretic peptide).
